# Tetanus Health Information on YouTube and TikTok: Cross-Sectional Analysis of Quality, Reliability, and Public Health Implications

**DOI:** 10.2196/85397

**Published:** 2026-06-24

**Authors:** Rui Huang, Yajing Shen, Guangqing Zou, Jun Zhou, Xiaolu Deng, Qian Li, Xiaoxiong Chen

**Affiliations:** 1 Department of Emergency Medicine, The First Affiliated Hospital (Southwest Hospital) of Army Medical University, No. 30, Gaotanyan Zheng Street, Shapingba District, Chongqing, China, 86 15696628112

**Keywords:** content analysis, health communication, infodemiology, reliability, social media, tetanus, TikTok, video quality, YouTube

## Abstract

**Background:**

Tetanus is a severe but vaccine-preventable neurological disease that remains a public health concern, especially in resource-limited settings. As social media becomes an important source of health information, concerns persist regarding the quality and reliability of tetanus-related content online.

**Objective:**

This study aimed to evaluate the quality, reliability, and thematic characteristics of tetanus-related videos on YouTube and TikTok and to examine the relationship between engagement metrics and information quality.

**Methods:**

A cross-sectional study was conducted using tetanus-related videos retrieved from YouTube and TikTok on August 1, 2025. The top 100 eligible videos from each platform were included (n=200). Video quality was assessed using the Global Quality Scale, whereas reliability and transparency were evaluated using the modified DISCERN tool and the *Journal of the American Medical Association* benchmark criteria. A thematic content analysis based on predefined coding categories was also performed. Video characteristics, source types, and engagement metrics were also collected. Spearman correlation analysis was used to examine associations between engagement indicators and quality scores.

**Results:**

YouTube videos showed significantly higher quality and reliability than TikTok videos, with higher median Global Quality Scale, modified DISCERN, and *Journal of the American Medical Association* scores (all *P*<.001). Compared with TikTok, YouTube videos more frequently discussed symptoms (92% vs 81%, *P*=.02), prevention (95% vs 78%, *P*<.001), treatment (88% vs 70%, *P*=.002), and wound management (77% vs 38%, *P*<.001). Lower-quality videos commonly contained incomplete prevention information, vague symptom descriptions, and limited source attribution. Videos produced by official medical organizations and professional health care creators generally achieved higher quality scores. Although engagement indicators were strongly correlated with each other, their associations with informational quality were relatively limited.

**Conclusions:**

YouTube provided more comprehensive and reliable tetanus-related information than TikTok, although content quality on both platforms remained inconsistent. Greater involvement from health care professionals and clearer evidence-based communication may help improve the quality of health information shared on social media platforms.

## Introduction

Tetanus is a severe neurological disease caused by *Clostridium tetani*, a spore-forming bacterium commonly found in soil and the gastrointestinal tracts of animals. It produces tetanus neurotoxin, a potent toxin that disrupts inhibitory neurotransmission and leads to muscle spasms and autonomic dysfunction [1]. Although effective vaccines and improved clinical management are available, tetanus continues to cause substantial morbidity and mortality worldwide [2]. The burden is particularly high in settings with limited health care resources, where access to vaccination and timely treatment may be restricted [3]. Recent reports from the World Health Organization indicate that 21,830 cases were documented globally in 2023, although the actual number is likely higher due to underreporting [4]. It is estimated that tetanus still accounts for tens of thousands of deaths each year [5]. These patterns suggest that, despite being preventable, tetanus remains an ongoing public health challenge, highlighting the need for better awareness and more effective health communication.

With the widespread use of the internet, social media platforms such as YouTube and TikTok have become important channels through which the public accesses health information [6]. Approximately 80% of internet users seek health information online. YouTube, a widely used video-sharing platform that lacks formal peer review mechanisms for uploaded content, was accessed by 73% of adults and 91% of individuals aged 15‐29 in the United States from 2019 to 2020 [7]. During and after the COVID-19 pandemic, YouTube experienced substantial growth in the consumption of health-related content, with reports indicating a 75% increase in views of health and news videos compared with the prepandemic period [8]. Although these platforms enable rapid dissemination and broad accessibility of health information, concerns remain regarding the accuracy, reliability, and completeness of online content. In addition, online engagement has increasingly been linked to research visibility through alternative metrics (Altmetrics), suggesting that digital dissemination may also influence the broader impact and public reach of health information [9,10].

The widespread use of video-based health information also raises concerns regarding health literacy. Major organizations, including the National Institutes of Health, the US Department of Health and Human Services, and the American Medical Association, recommend that patient education materials be written at or below a sixth-grade reading level [11]. However, many online resources exceed this level, potentially limiting accessibility and comprehension.

Infodemiology provides a useful framework to evaluate the distribution and quality of online health information [12]. Although prior studies have assessed social media content across various health topics, no study has systematically evaluated tetanus-related videos on major platforms. By integrating content quality assessment with engagement and dissemination metrics, this study contributes to the core objectives of infodemiology, offering insights into how health information is structured, spread, and interpreted within contemporary digital environments.

Therefore, this study aimed to assess the quality, reliability, and content characteristics of tetanus-related videos on YouTube and TikTok. Specifically, we aimed to (1) evaluate video quality and reliability using the Global Quality Scale (GQS) [13], the *Journal of the American Medical Association* (*JAMA*) benchmark criteria [14], and the modified DISCERN (mDISCERN; originally derived from the DISCERN instrument: Quality Criteria for Consumer Health Information on Treatment Choices) [15]; (2) analyze thematic content characteristics of the videos; (3) compare engagement metrics and their association with information quality across platforms; and (4) identify gaps to inform improved digital health communication strategies. The instruments mentioned in (1) have been validated in previous studies [16,17].

## Methods

### Ethical Considerations

This study did not involve human participants, clinical data, biological specimens, or laboratory animals. All data were obtained from publicly accessible video content on YouTube and TikTok. No identifiable personal information was collected. Therefore, institutional review board approval was not required.

### Study Design

This cross-sectional study examined the quality, reliability, and dissemination characteristics of tetanus-related videos on YouTube and TikTok. The aim was to evaluate how these platforms present health information and identify potential gaps in online health communication.

#### Search Strategy and Data Collection

Videos were identified on August 1, 2025, using the keyword “tetanus” on YouTube and TikTok. Data collection was conducted on a single day to provide a cross-sectional snapshot of available content and reduce potential variation caused by changes in platform algorithms over time.

To reduce personalization bias, all searches were performed using newly created accounts with no prior viewing history or interactions. Browser cache and search history were cleared before data collection to further minimize algorithmic influence on search results. Videos were retrieved using the default platform ranking, and the top 100 most relevant videos from each platform were included (n=200). All selected videos were recorded and archived to ensure reproducibility.

### Eligibility Criteria

Eligibility criteria were defined to ensure that the included videos were relevant, comparable, and aligned with the study objective of evaluating publicly accessible tetanus-related health information.

Videos were eligible for inclusion if they met the following criteria: (1) addressed tetanus-related education, prevention, or management; (2) were presented in English; (3) had a duration between 20 seconds and 20 minutes; and (4) had been available online for at least 10 days prior to data collection. Videos were excluded if they (1) were commercial advertisements or promotional materials, to avoid a noneducational bias; (2) were duplicates or reposted content, to prevent redundancy; (3) were not directly related to tetanus health information, to maintain thematic consistency. These criteria ensured that the final sample reflected meaningful, nonredundant, and publicly relevant educational content.

### Video Categorization

Each video was categorized by its source to capture differences in credibility and educational approach. Categories included (1) medical official accounts (eg, hospitals, public health agencies), (2) nonmedical official accounts (eg, news media, science organizations), (3) professional medical creators (eg, doctors, nurses, medical educators), (4) nonmedical professional creators (eg, science communicators, educators), and (5) the general public (eg, personal experiences, lay users).

### Evaluation Instruments

Videos were independently evaluated by two emergency physicians with expertise in tetanus prevention. Prior to formal assessment, both reviewers underwent standardized training to ensure consistent application of the scoring criteria.

Three validated instruments were used: (1) the mDISCERN tool, which assesses the reliability and quality of health information; (2) the GQS, which evaluates the overall educational quality and usefulness of the content; and (3) the *JAMA* benchmark criteria, which assess transparency based on authorship, attribution, disclosure, and currency.

All videos were scored strictly in accordance with the original published criteria for each instrument [13-15]. Detailed scoring frameworks have been extensively described in previous studies and were not repeated here to avoid redundancy.

Each video was independently rated by both reviewers, and the final score was calculated as the mean of the two assessments. Discrepancies were resolved through discussion until consensus was reached.

### Thematic Content Analysis

In addition to quantitative quality assessment, a thematic content analysis was conducted to examine the informational characteristics of the videos. The coding framework was informed by prior infodemiology literature, commonly evaluated dimensions of online health information quality, and clinically relevant aspects of tetanus prevention and management. The predefined thematic categories included [18,19]: (1) disease overview, (2) symptoms, (3) prevention (eg, vaccination), (4) treatment, (5) wound management, and (6) misinformation or incomplete information.

Misinformation was defined as content that was inconsistent with established medical evidence or lacked essential information required for safe and accurate understanding. Two reviewers independently coded all videos according to this framework. Each thematic category was recorded as present or absent for each video. Disagreements were resolved through discussion and consensus. Intercoder reliability for thematic classification was assessed using Cohen κ statistics. In addition to structured coding, reviewers noted recurring features observed in lower-quality videos, such as incomplete or oversimplified explanations, potentially misleading statements, limited or absent supporting evidence, and lack of clear source attribution. For interpretive purposes, videos were generally considered to be of lower quality when they received relatively low scores on the applied evaluation tools (eg, GQS ≤2 and/or mDISCERN ≤1) and/or when such issues were identified during qualitative review. This approach enabled us to reflect not only measurable differences in scoring but also practical limitations in how health information was presented to viewers. The relationship between account type and thematic content was also descriptively examined to explore whether different sources emphasized distinct types of health information.

### Data Extraction

For each video, we collected descriptive and engagement-related variables, including number of likes, comments, shares, favorites, views, followers, upload date, video duration, and account verification status. The geographical origin of the account was also recorded to explore potential regional differences in content distribution.

#### Statistical Analysis

All statistical analyses were performed using GraphPad Prism (version 10.1). Data normality was assessed prior to analysis. Categorical variables were expressed as frequencies and percentages, while nonnormally distributed continuous variables were presented as medians and interquartile ranges (IQR). Between-group comparisons of continuous variables were conducted using the Mann-Whitney *U* test for nonnormally distributed data and the independent-samples *t* test for normally distributed data, as appropriate. Categorical variables were compared using the *χ*^2^ test or Fisher exact test, as appropriate. Spearman correlation coefficients were calculated to examine relationships between engagement metrics and professional quality scores. All statistical tests were 2-tailed, and a *P* value <.05 was considered statistically significant.

## Results

### Video Characteristics

A total of 200 videos met the inclusion criteria, comprising 100 from YouTube and 100 from TikTok. Overall, YouTube videos were significantly longer in duration compared with TikTok videos (*P*<.001). Basic video characteristics and engagement metrics are summarized in Table 1.

**Table 1.** Basic characteristics of the videos.

Information	TikTok	YouTube	*P* value
Likes (IQR)	642 (89.25-4538)	262.5 (27.25-1493)	.98
Comments (IQR)	44 (8.25-291)	9.5 (1.00-52.75)	.19
Favorites (IQR)	87.5 (14.25-503.8)	Not applicable	.004
Views (IQR)	75,350 (10475-619,275)	15,693 (2584-72,514)	.49
Followers (IQR)	11,500 (1314-93,870)	38,950 (3153-343,500)	.23
Duration (s) (IQR)	38.5 (25-74.75)	78.5 (45.25-346)	<.001
Days since posting (IQR)	403 (163.5-692)	483 (258.8-867)	.09
Global Quality Scale score (IQR)	2 (1-3）	3 (3-4）	<.001
Modified DISCERN score (IQR)	1 (1-2）	2 (2-3）	<.001
*Journal of the American Medical Association* score (IQR)	1 (1-1）	2 (1-2）	<.001

### Quality and Reliability Scores

**Figure 1.**
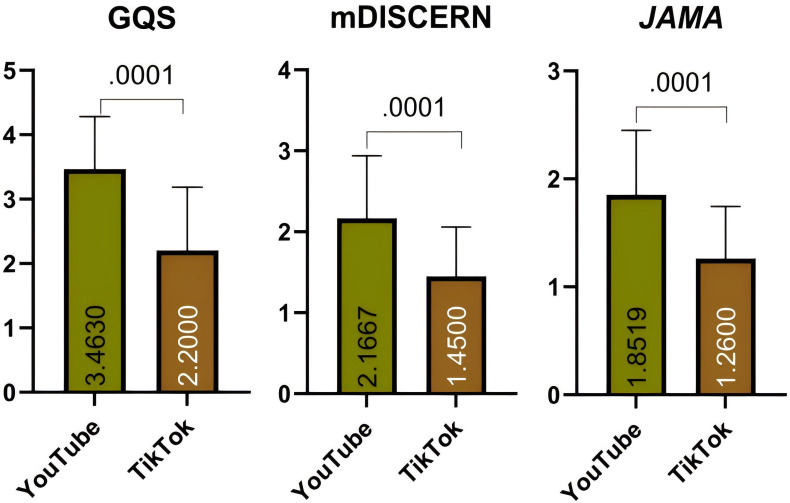
Comparison of video quality and reliability scores between YouTube and TikTok. Box plots show median values and interquartile ranges for Global Quality Scale (GQS), modified DISCERN (mDISCERN), and *Journal of the American Medical Association* (*JAMA*) benchmark scores. YouTube videos demonstrated significantly higher scores across all three instruments (all *P*<.001).

**Figure 2.**
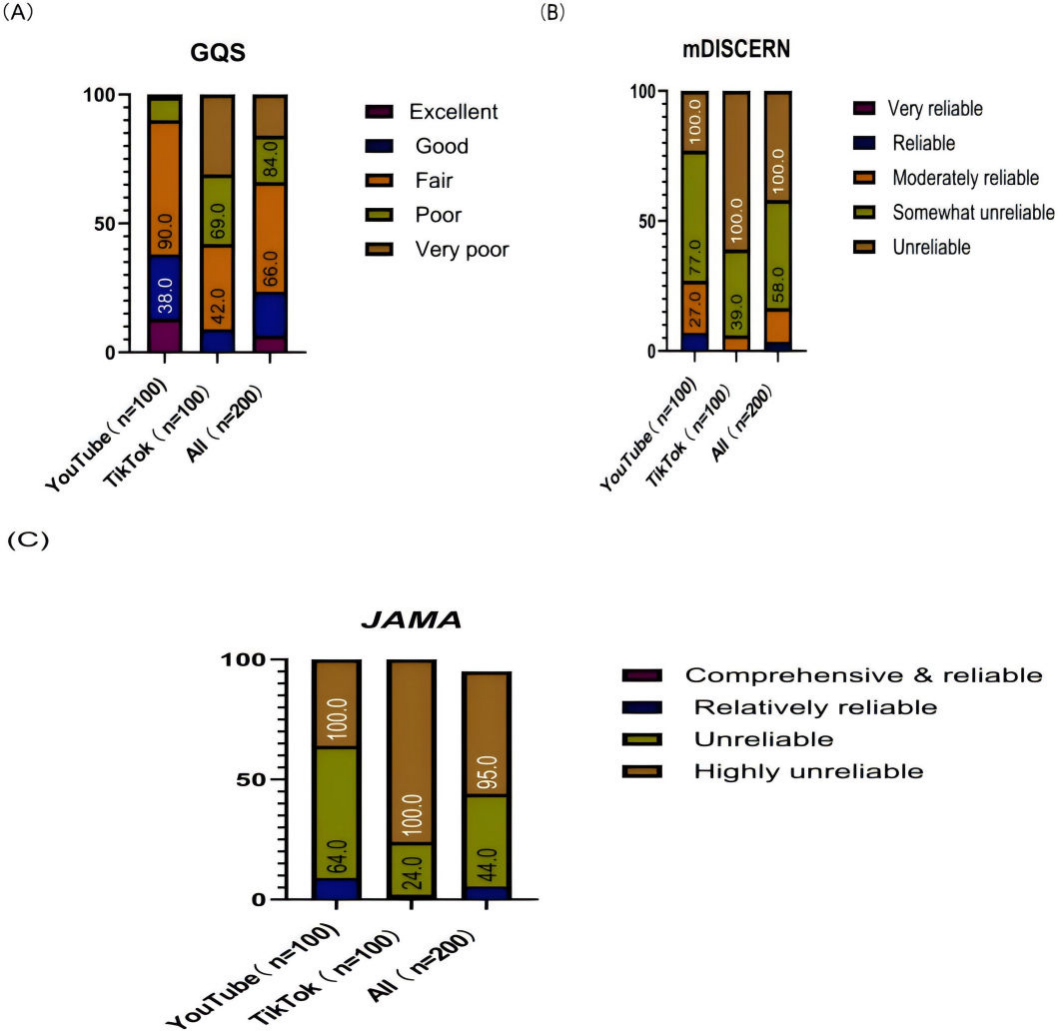
Overall distribution of video quality and reliability levels across platforms. Although YouTube videos showed higher scores than TikTok videos, the majority of videos on both platforms were of moderate to low quality and reliability. GQS: Global Quality Scale; *JAMA*: *Journal of the American Medical Association*; mDISCERN: modified DISCERN.

Across all three evaluation tools, YouTube videos demonstrated significantly higher quality and reliability than those on TikTok (Figure 1). The median GQS score was 3 (IQR 2‐4) for YouTube and 2 (IQR 1‐3) for TikTok (*P*<.001). Similarly, the median mDISCERN score was 2 (IQR 1‐3) for YouTube and 1 (IQR 0‐2) for TikTok (*P*<.001). *JAMA* benchmark scores followed the same pattern, with median scores of 2 for YouTube and 1 for TikTok (*P*<.001). Overall, while YouTube content showed better educational quality, videos from both platforms were generally of moderate to low reliability (Figure 2).

### Quality and Reliability by Source

**Figure 3.**
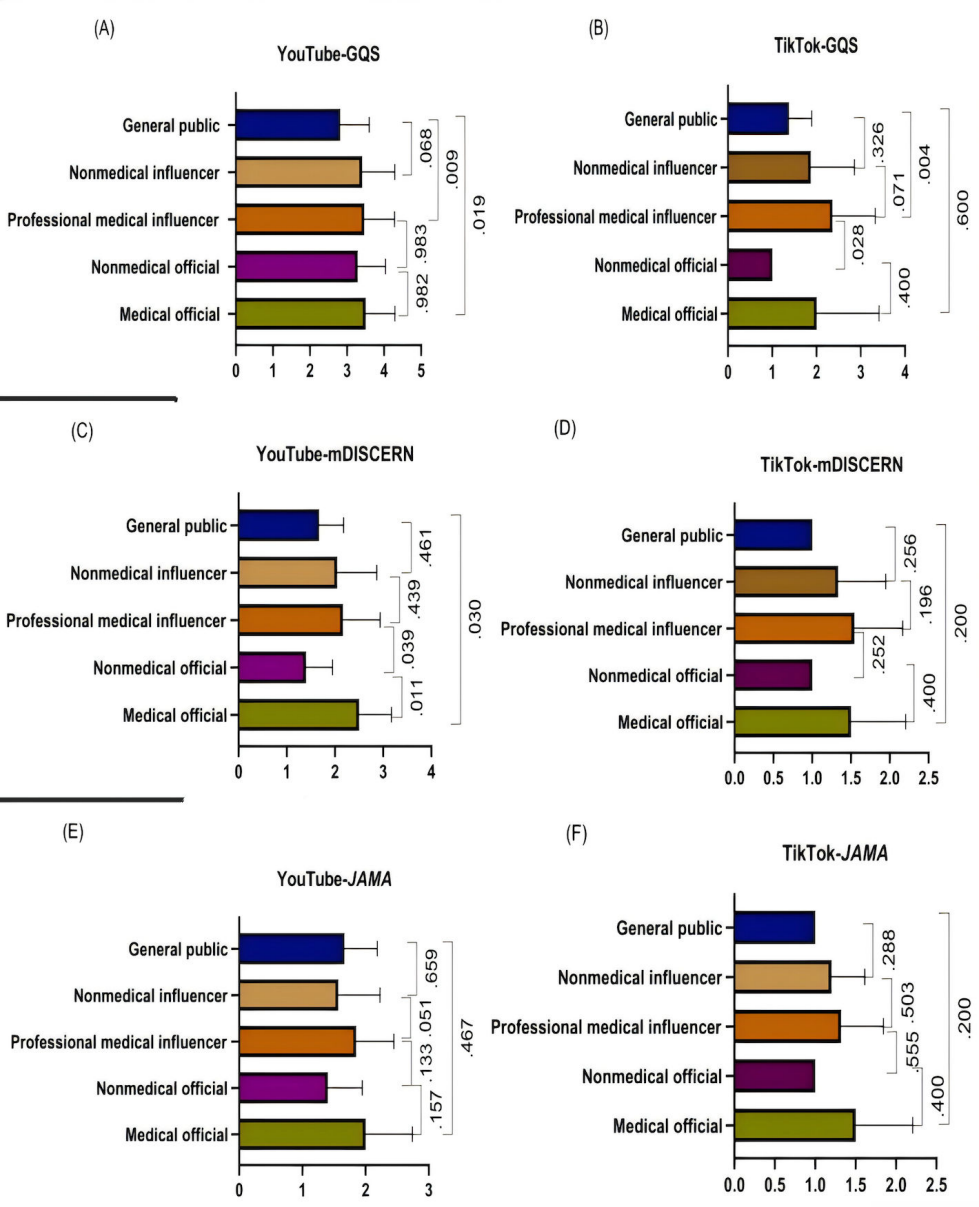
Quality and reliability scores stratified by source of video upload. Videos from official medical organizations and professional medical creators demonstrated higher Global Quality Scale (GQS), modified DISCERN (mDISCERN), and *Journal of the American Medical Association* (*JAMA*) benchmark scores compared with other sources. Distribution patterns differed between YouTube and TikTok.

The quality and reliability of videos varied considerably based on their source (Figure 3). On YouTube, videos from official medical organizations (n=12, 12%) achieved the highest median scores across all instruments, followed by content produced by professional medical creators (n=54, 54%). In contrast, TikTok content was predominantly produced by professional medical creators (n=72, 72%), who also achieved the highest quality scores on that platform. There was no significant difference in *JAMA* scores between platforms (*P*>.05). These findings highlight the role of professional oversight and expertise in producing trustworthy educational content. Differences in thematic emphasis were also observed across account types. Videos uploaded by official medical organizations and health care professionals more commonly focused on prevention, vaccination, wound management, and treatment-related information. These videos were generally more structured and were more likely to include evidence-based recommendations or references to clinical care.

In contrast, videos posted by nonmedical creators more often emphasized brief symptom descriptions, personal experiences, or general awareness messages, with less detailed discussion of prevention or management. Simplified explanations and omission of key clinical information were more frequently observed in these videos. On TikTok in particular, short-form videos from noninstitutional accounts tended to prioritize concise delivery and audience engagement over informational depth.

### Thematic Content Characteristics of Tetanus-Related Videos

Intercoder agreement for thematic classification was substantial to almost perfect across the predefined thematic categories. Cohen κ values were 0.703 for disease overview, 0.826 for symptoms, 0.831 for prevention, 0.887 for treatment, 0.908 for wound management, and 0.858 for misinformation or incomplete information.

Thematic content analysis revealed significant differences in the distribution of informational components between platforms (Table 2). Videos on YouTube more frequently addressed symptoms (n=92, 92% vs n=81, 81%, *P*=.02), prevention (n=95, 95% vs n=78, 78%, *P*<.001), treatment (n=88, 88% vs n=70, 70%, *P*=.002), and wound management (n=77, 77% vs n=38, 38%, *P*<.001) compared to TikTok videos. In contrast, there were no statistically significant differences between platforms in the inclusion of general disease overview (n=95, 95% vs n=93, 93%, *P*=.55) or the presence of misinformation or incomplete information (n=9, 9% vs n=12, 12%, *P*=.50).

**Table 2.** Thematic content characteristics of tetanus-related videos on YouTube and TikTok (N=200).

Characteristic	YouTube (n=100), n (%)	TikTok (n=100), n (%)	*P* value
Disease overview	95 (95)	93 (93)	.55
Symptoms	92 (92)	81 (81)	.02
Prevention	95 (95)	78 (78)	<.001
Treatment	88 (88)	70 (70)	.002
Wound management	77 (77)	38 (38)	<.001
Misinformation or incomplete information	9 (9)	12 (12)	.50

To complement these findings, qualitative review identified several recurring patterns in lower-quality videos. Some videos presented overly simplified or incomplete explanations of tetanus, particularly regarding symptoms and prevention. For instance, wound care was sometimes described as sufficient on its own, without reference to vaccination as the primary preventive measure. In other cases, symptom descriptions were vague or imprecise, such as attributing muscle stiffness to general fatigue without explaining its neurological basis. In addition, missing source attribution and the absence of clear, actionable guidance were common. These features were observed on both platforms and were more frequently seen in videos with lower-quality scores.

### Publication Timeline and Account Verification

**Figure 4.**
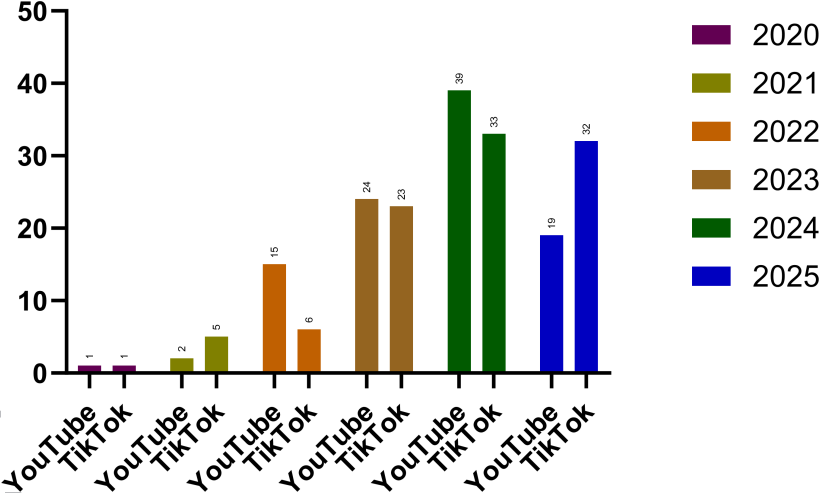
Distribution of tetanus-related videos by year (2020–2025) on YouTube and TikTok. Most videos were uploaded in 2024 on both platforms.

Videos analyzed were uploaded between 2020 and 2025, with the majority posted in 2024 (YouTube: n=39, 39%; TikTok: n=33, 33%) (Figure 4). Account verification was more common on YouTube (n=20, 20%) than on TikTok (n=3, 3%), suggesting differences in platform moderation and credibility indicators.

### Geographical Distribution of Video Sources

The geographic distribution of video sources varied across platforms. On YouTube, most videos originated from India (n=40, 40%) and the United States (n=31, 31%), followed by Indonesia (n=4, 4%) and Pakistan (n=4, 4%). On TikTok, the distribution was more dispersed, with India (n=19, 19%) and the United States (n=15, 15%) representing the largest shares. When excluding videos with unspecified origins, India, the United States, and Indonesia accounted for the majority of content on both platforms.

### Engagement Metrics and Correlation Analysis

**Figure 5.**
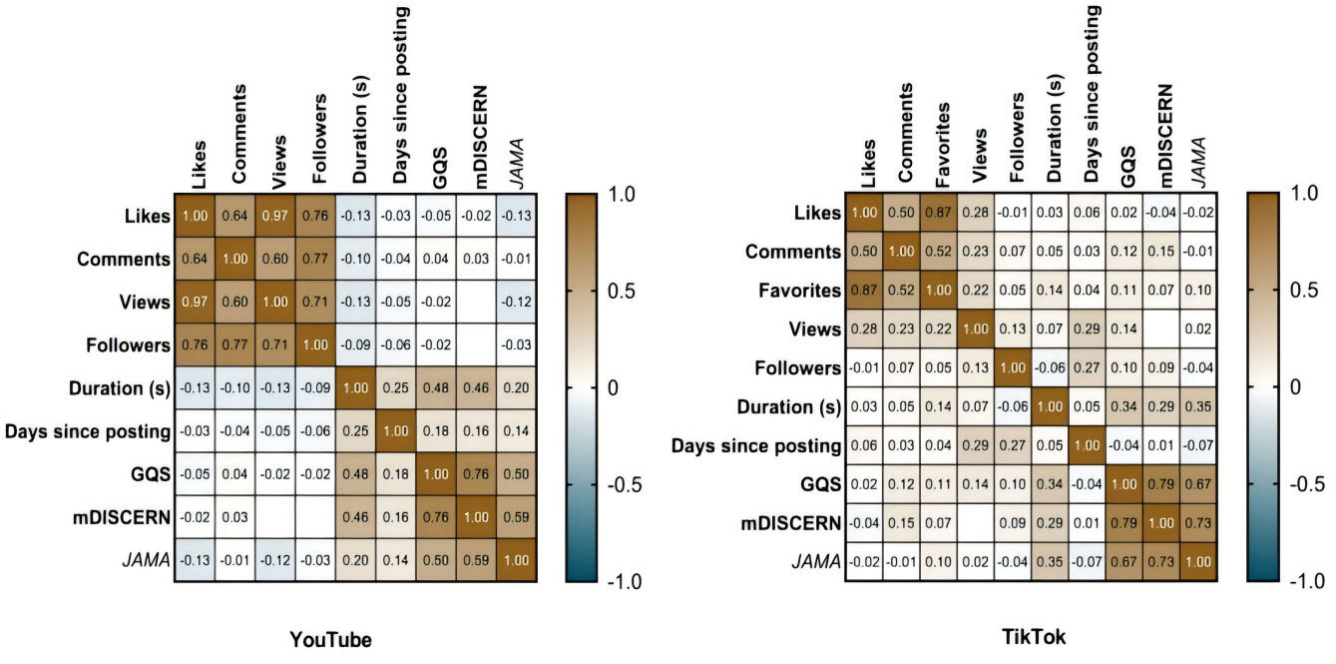
Correlations between engagement metrics and quality scores. Scatter plots show the relationships between engagement indicators (likes, views, favorites) and GQS, mDISCERN, and *JAMA* scores. Engagement metrics were strongly correlated with each other, whereas their correlations with quality scores were weaker. GQS: Global Quality Scale; *JAMA*: *Journal of the American Medical Association*; mDISCERN: modified DISCERN.

Engagement metrics showed strong interrelationships, particularly between likes and views on YouTube (*r*=0.97, *P*<.001) and between likes and favorites on TikTok (*r*=0.87). However, the correlation between engagement indicators and quality scores was moderate, suggesting that highly interactive content does not always align with higher educational value. For example, on YouTube, GQS scores correlated moderately with mDISCERN (*r*=0.76) and *JAMA* (*r*=0.50) scores, while on TikTok, GQS correlated strongly with mDISCERN (*r*=0.79) and *JAMA* (*r*=0.67) scores. Notably, video duration showed only a weak positive correlation with quality scores (YouTube: *r*=0.48; TikTok: *r*=0.34), indicating that while longer videos may provide more comprehensive information, length alone does not guarantee higher educational quality (Figure 5).

## Discussion

### Principal Findings

From an infodemiology perspective, this study provides a systematic evaluation of how health information is generated, disseminated, and consumed on social media platforms, highlighting key challenges in digital health communication. This study evaluated tetanus-related videos on YouTube and TikTok in terms of quality, reliability, and content characteristics. Across all evaluation tools, YouTube videos had higher scores than TikTok videos. However, the overall quality on both platforms remained limited, with most videos falling within the low-to-moderate range.

### Platform Differences in Quality and Video Characteristics

Differences between platforms may be partly related to video format. In this study, YouTube videos were generally longer, which allows more space to explain clinical concepts. In contrast, TikTok videos were shorter and more condensed, which may restrict the amount of information presented.

### Thematic Content Differences

Thematic analysis showed clear differences in content coverage. YouTube videos more frequently included key clinical topics such as symptoms, prevention, treatment, and wound management. These elements are essential for tetanus prevention and early management. TikTok videos less often covered these components, particularly wound management, where the gap between platforms was most pronounced. Despite these differences, the proportion of videos containing misinformation or incomplete information did not differ significantly, suggesting that accuracy issues exist on both platforms.

### Implications of Low-Quality Content

Inaccurate or vague descriptions of symptoms may further reduce the educational value of these videos, especially for conditions such as tetanus where early recognition is important. In addition, the lack of source attribution and absence of clear recommendations suggest limited transparency and may undermine the credibility of the information presented.

Taken together, these findings indicate that the challenge extends beyond overt misinformation to include the omission of essential clinical details. Similar patterns have been reported in previous evaluations of online health information, where incomplete or poorly contextualized content reduced overall reliability [20]. This may partly reflect the constraints of short-form content formats and the tendency to prioritize simplicity and engagement over informational depth [21].

Improving the clarity, accuracy, and completeness of key health messages should therefore be a priority for enhancing the effectiveness of social media–based health communication [22].

### Role of Content Source

Videos produced by health care professionals and medical organizations tended to have higher quality scores. This pattern has been reported in previous studies and reflects the importance of professional involvement [23]. At the same time, variation within this group indicates that expertise alone does not guarantee high-quality communication, especially when content is adapted for social media formats. Thematic differences across account types may also help explain the variability in video quality observed in this study. Videos produced by health care professionals or institutional accounts more frequently focused on prevention, vaccination, and wound management, whereas nonmedical creators tended to emphasize brief symptom descriptions or general awareness content [24].

This pattern suggests that professional background influences not only overall information quality but also the depth and focus of health communication. On short-form platforms in particular, content creators may prioritize concise delivery and audience engagement, which can result in the omission of clinically important details.

### Engagement and Information Quality

Engagement indicators such as likes and views were strongly correlated with each other but only moderately associated with quality scores. This suggests that widely viewed content is not necessarily more reliable. The discrepancy between popularity and informational value may contribute to the spread of incomplete or oversimplified health information.

### Comparison With Previous Studies

These findings are consistent with prior research on online health information [25,26]. Studies evaluating YouTube content across different medical topics have reported substantial variability in quality and frequent gaps in completeness and reliability [27,28]. Systematic reviews have similarly shown that health-related videos often lack key clinical information despite high visibility [20]. Comparative studies have also suggested that short-form video platforms may further limit informational depth [29,30]. The present study extends these observations to tetanus-related content, particularly in areas such as prevention and wound management. These findings contribute directly to the growing field of infodemiology by identifying gaps in the quality and dissemination of online health information, with implications for improving digital health communication strategies. Similar concerns regarding the readability and quality of online health information have also been reported in non-English settings, including Turkish-language patient education materials [31], suggesting that variability in accessibility and reliability may represent a broader international challenge in digital health communication.

In recent years, artificial intelligence (AI)–assisted information systems and conversational chatbots have increasingly been used as sources of health information. Previous studies have shown that AI-generated medical content may improve accessibility and user engagement, although concerns remain regarding readability, reliability, and clinical accuracy [32]. Compared with AI-generated responses, social media videos may provide more intuitive and visually accessible information, but they can also contain oversimplified or incomplete medical explanations. These issues suggest that maintaining the quality and consistency of online health information remains challenging across different digital platforms.

### Public Health Implications

Tetanus is a preventable disease, and accurate information on vaccination and wound care is essential [33]. Social media platforms provide broad access to such information [34,35], but the variability in quality observed in this study indicates that current content may not fully support effective prevention.

Improving content quality may require greater involvement from health care professionals, clearer credibility indicators, and platform-level strategies to promote reliable information [36]. Adapting content to platform characteristics while maintaining accuracy is also important.

### Strengths

This study evaluated tetanus-related videos on both YouTube and TikTok using multiple validated assessment tools, including GQS, mDISCERN, and the *JAMA* benchmark criteria. By combining quality evaluation with thematic analysis and engagement metrics, the study provides a broader view of how tetanus-related health information is presented and disseminated on social media platforms. In addition, the inclusion of two widely used video platforms improves the practical relevance of the findings within current digital health communication settings.

### Limitations

Several limitations should be acknowledged. First, only English-language videos were included, which may limit the generalizability of the findings to other linguistic and cultural contexts. Second, data collection was performed at a single time point, whereas social media content and platform algorithms may change over time. Although newly created accounts and search-history controls were used to reduce personalization bias, algorithmic influence on search results could not be completely eliminated. Finally, because of the cross-sectional design, the study was unable to evaluate causal relationships between video characteristics, user engagement, and information quality.

### Conclusions

YouTube generally provided more comprehensive and reliable tetanus-related information than TikTok, although the overall quality of videos on both platforms remained variable. Videos produced by health care professionals or institutional accounts were more likely to include information on prevention, vaccination, treatment, and wound management. Highly viewed videos were not always associated with better informational quality.

Many videos contained incomplete explanations or lacked important clinical details, which may limit their educational value for the public. Given that tetanus is largely preventable, improving the clarity, accuracy, and transparency of online health information remains important. Greater participation from qualified health care professionals and improved content oversight may help improve the quality of health communication on social media platforms.

Although AI-assisted tools may improve access to health information, they should currently be regarded as supplementary resources rather than replacements for professional medical advice. Care should be taken when interpreting online medical information that may be incomplete, oversimplified, or insufficiently evidence-based.

## Supplementary material

10.2196/85397 Multimedia Appendix 1 Raw data.

## Abbreviations

AI: artificial intelligence
GQS: Global Quality Scale
*JAMA*: *Journal of the American Medical Association*
mDISCERN: modified DISCERN
WHO: World Health Organization
